# Effect of supplementation with *Glycyrrhiza uralensis* extract and *Lactobacillus acidophilus* on growth performance and intestinal health in broiler chickens

**DOI:** 10.3389/fvets.2024.1436807

**Published:** 2024-07-18

**Authors:** Ximei Li, Jiawei Li, Haotian Yuan, Yan Chen, Shuaibing Li, Susu Jiang, Yingpai Zha Xi, Guohua Zhang, Jianxiong Lu

**Affiliations:** ^1^College of Life Science and Engineering, Northwest Minzu University, Lanzhou, China; ^2^Department of Animal Science and Technology, Gansu Agriculture Technology College, Lanzhou, China

**Keywords:** *Glycyrrhiza uralensis* extract, *Lactobacillus acidophilus*, growth performance, intestine health, broiler chickens

## Abstract

Intestinal microbiota community is an important factor affecting the nutritional and health status of poultry, and its balance is crucial for improving the overall health of poultry. The study aimed to investigate the effect of dietary supplementation with *Glycyrrhiza uralensis* extract (GUE), *Lactobacillus acidophilus* (Lac) and their combination (GL) on growth performance and intestinal health in broilers in an 84-day feeding experiment. Supplementary 0.1% GUE and 4.5×10^7^ CFU/g Lac significantly increased average daily gain (ADG), and GL (0.1% GUE and 4.5×10^7^ CFU/g Lac) increased ADG and average daily feed intake (ADFI), and decreased feed conversion rate (FCR) in broilers aged 29 to 84 d and 1 to 84 d. Dietary GUE, Lac and GL increased the superoxide dismutase (SOD) and glutathione peroxidase (GSH-PX) activity and decreased Malondialdehyde (MDA) content in the jejunum mucosa of broilers, and increased secretory IgA (sIgA) content in broilers at 84 d. Moreover, GUE, Lac and GL increased cecal microbial richness and diversity, and modulated microbial community composition. Both GUE and Lac reduced the harmful bacteria *Epsilonbacteraeota*, *Helicobacter*, and *H. pullorum* at 28 d and *Proteobacteria*, *Escherichia*, and *E. coli* at 84 d, while Lac and GL increased beneficial bacteria *Lactobacillus* and *L. gallinarum* at 28 d. Compared with individual supplementation, GL markedly increased the SOD activity and the sIgA content, and reduced *Helicobacter* and *Helicobacter pullorum*. In conclusion, GUE and *Lactobacillus acidophilus* as feed additives benefit growth performance and intestinal health, and their combined use shows an even more positive effect in broilers.

## 1 Introduction

With the continuous improvement of the scale and intensity of poultry farming, the production performance of poultry has been gradually improved. However, broilers are susceptible to external factors such as disease, nutrition, and environment due to the imperfect development of intestinal function and low immunity, which frequently result in poor health, enhanced stress response, and intestinal imbalance (1). In the era of a complete ban on antibiotics as feed additives and a focus on healthy breeding, the development and utilization of feed additives like medicinal plant extracts and probiotics have garnered much attention (2, 3).

*Glycyrrhiza uralensis* Fisch is a traditional medicinal and edible plant with a long history of dietary and pharmacological applications, and its edible and medicinal parts are the root and rhizome (4, 5). The primary active ingredients in *Glycyrrhiza uralensis* extract (GUE) include glycyrrhiza polysaccharides, triterpene saponins (glycyrrhizic acid, glycyrrhizinic acid, etc.), and flavonoids (chalcone, isoflavone, etc.) (6). GUE exhibits various pharmacological effects, such as anti-inflammatory (7), antioxidant (8), antiviral (9), immune regulation (10) and improvement intestinal microbiota (11). *Lactobacillus acidophilus* is a dominant microbiota in the gastrointestinal tract (GIT) of humans and animals, often used as a probiotics due to its health-promoting properties (12). Probiotics *Lactobacillus* is typically employed as Direct-Fed microbiota to poultry and other livestock to enhance intestinal health (13), enhancing immunity (14, 15) and reducing colonization of pathogens in the GIT (16).

The intestinal tract is a crucial organ for digestion and absorption of feed nutrients in animals, closely linked to a range of physiological and biochemical processes, contributing to the animal’s nutrition and overall health (17). The intestinal microbiota is critical for maintaining host intestinal health, acting as the organism’s “second brain,” preventing pathogen colonization, modulating the intestinal epithelial barrier and inflammatory response (18, 19). The colonization of gut microbiota is relatively stable, and their composition and structure are influenced by factors such as diet, age, and feeding methods in animals (20). Changes in the GIT microbial community impact feed efficiency, productivity, and the health of chickens (21). The cecum, with the highest microbial colonization and the most abundant and diverse microbial community in the broiler gut, is crucial for the overall performance in poultry (22, 23). Generally, the chyme stays in the cecum for an extended period, allowing thorough decomposition of many feed ingredients by microbiota to improve nutrient utilization (24). Therefore, the balance of cecum microbiota is essential for enhancing overall performance of poultry.

Several recent studies have confirmed that GUE (25, 26) and probiotics (27, 28) promote growth and improve intestinal health in broilers. However, the potential effects of the combination of GUE and *Lactobacillus* on growth performance and intestinal health are not yet fully understood. Thus, the purpose of the present study was to investigate the impact of GUE, *Lactobacillus acidophilus* and their combination on growth performance and intestinal health in *Liangfenghua* broiler chickens, a medium-growing broiler strain known for its popularity due to the excellent meat quality. The findings of this study would be beneficial in comprehending the intestinal microecology of chickens and offering novel perspectives on the upkeep of intestinal wellness in broiler chickens that are fed antibiotic-free diets.

## 2 Materials and methods

### 2.1 Ethics statement

All procedures involving in animals were performed following the Regulations for the Administration of Affairs Concerning Experimental Animals (Ministry of Science and Technology, China, 2004) and were approved and supervised by the Northwest Minzu University Animal Care and Use Committee (Permit No. xbmu-sm-20210130).

### 2.2 Experimental design and animal management

The *Glycyrrhiza uralensis* extract (GUE; prepared from the root of GU using decoction extraction and ethanol precipitation methods; Yalan Pharmaceutical Co, Gansu, China) and the *Lactobacillus acidophilus* (Lac; Zhongxin Bio-Technology Co., Hebei, China; 3 × 10^9^ CFU/g) used in the present study were commercial products.

A total of 420 healthy one-day-old male *Liangfenghua* broiler chickens were randomly allocated into 4 dietary treatments, each with 7 replicates and 15 chickens per replicate. The treatment groups included: (1) basal diet (Con group); (2) basal diet supplemented with 0.1% GUE (GUE group); (3) basal diet supplemented with Lac at 4.5 × 10^7^ CFU/g (Lac group); and (4) basal diet supplemented with 0.1% GUE and 4.5 × 10^7^ CFU/g Lac (GL group). Table 1 presented the composition and nutritional levels of the basal diet, formulated in accordance with the “Broilers Feeding Standard in China” (NY/T 33-2004). The trial was conducted at Gansu Agricultural Vocational Farm Co. in Gansu, China.

**Table 1:** Composition and nutrient levels of basal diets (air-dry basis).

Items	Content	
	1 to 28 d of age	29 to 84 d of age
**Ingredients (%)**		
Corn	55.00	57.50
Soybean oil	2.90	4.20
Soybean meal	29.00	24.00
Cottonseed meal	1.40	1.70
Rapeseed meal	1.90	3.00
Corn gluten meal	6.80	6.60
CaHPO_4_	1.80	2.50
NaCl	0.78	0.08
*L*-Lys•HCL	0.15	0.11
*DL*-Met	0.00	0.08
Cys-Cys	0.07	0.03
Premix^a^	0.20	0.20
Total	100.00	100.00
**Nutrient levels**^b^		
ME/(MJ/kg)	12.49	12.86
CP (%)	21.46	19.91
Ca (%)	0.90	0.86
TP (%)	0.68	0.65
Lys (%)	1.15	1.00
Met (%)	0.70	0.40

a The premix provided the following per kg of diets: 1 to 28 d of age, VA 12,000 IU, VD_3_ 3,500 IU, VE 60 IU, VK_3_ 4 mg, VB_1_ 2.5 mg, VB_2_ 10 mg, VB_6_ 6 mg, VB_12_ 8 μg, D-pantothenic acid 40 mg, nicotinic acid 75 mg, folic acid 10 mg, biotin 0.8 mg, choline 700 mg, Zn 90 mg, Fe 110 mg, Cu 20 mg, Mn 100 mg, I 0.5 mg, Se 0.3 mg; 29 to 84 d of age, VA 10,000 IU, VD_3_ 3,000 IU, VE 50 IU, VK_3_ 3.5 mg, VB_1_ 2 mg, VB_2_ 10 mg, VB_6_ 5 mg, VB_12_ 6 μg, D-pantothenic acid 20 mg, nicotinic acid 60 mg, folic acid 8 mg, biotin 0.6 mg, choline 600 mg, Zn 80 mg, Fe 100 mg, Cu 15 mg, Mn 80 mg, I 0.5 mg, Se 0.3 mg.

b Nutrient levels were all calculated values.

Before the experiment commenced, the chicken coops and internal equipment were cleaned and fumigated for disinfection. Chicks aged 1–28 days were reared in three-layer ladder cages (1.2 × 0.9 × 1.0 m, length × width × height), with the coop preheated before the chicks entering. During the first week, the coop temperature was maintained at 34°C with a relative humidity (RH) of 50%. Subsequently, the temperature was reduced by 2°C per week until reaching 26°C, with RH at 45%. From 29 to 84 days old, the broilers were raised on the ground, ensuring the coop remained dry, hygienic, and well-ventilated. All the chickens were kept in a single room comprising six floor pens, each measuring 300 × 350 cm. Each pen had solid white plastic walls and was divided by wire mesh into 10 compartments. These compartments were equipped with a round feeder pan (diameter = 30 cm) and one nipple drinker. Cork shavings were used as litter, and the litter was replaced every 3 days. The chickens were exposed to 12-h light/dark cycles daily throughout the test period and had *ad libitum* access to feed and water. The broilers were vaccinated with Newcastle disease vaccine and the infectious bursal polyvalent vaccine on d 7 and 14 of the experiment, respectively.

### 2.3 Growth performance determination

All broilers were weighed at 1, 28, and 84 days of age after a 12-h fast. The feed intake per replicate was recorded daily to calculate the average daily gain (ADG), average daily feed intake (ADFI) and feed conversion rate (FCR, feed/gain). Chicken mortality was recorded after which performance parameters were corrected for mortality.

### 2.4 Sample collection

All birds were fasted for 12 h (overnight) prior to collecting test samples. On d 28 and 84, two broilers with similar body weight per replicate from each group were selected and euthanized by severing the jugular vein. The abdomen was disinfected with 75% ethanol, then immediately dissected. The entire intestine was carefully removed from the abdominal cavity, and the jejunum and cecum were separated with a sterile scalpel. The middle part of jejunum was cut longitudinally, washed with 4% phosphate buffer solution (PBS), and jejunal mucosa was scraped with sterilized slides, put into RNAase-free tubes, snap-frozen in liquid nitrogen, and stored at −80°C for the determination of antioxidant indexes and secretory IgA (sIgA) content. The cecum contents were carefully collected, homogenized with a sterile spatula, transferred to CryoPure Tubes (Sarstedt AG + Co., Nümbrecht, Germany), snap-frozen in liquid nitrogen and stored at −80°C until they were processed for microbial DNA analysis.

### 2.5 Intestinal antioxidant and sIgA analysis

The activity of superoxide dismutase (SOD), glutathione peroxidase (GSH-PX), and the content of Malondialdehyde (MDA) in the jejunum mucosa were measured by assay kit (Shanghai Gantu Biotechnology Co., China). The level of intestinal sIgA was determined by double antibody one-step sandwich enzyme-linked immunosorbent assay (ELISA; Shanghai Liquid Quality Testing Technology Co., China). All detection methods were performed according to the manufacturer’s instructions.

### 2.6 Intestinal microbial diversity analysis

Bacterial genomic DNA was isolated from the cecal contents using the TGuide S96 kit (DP812; Tiangen Biotech Co., China) following the manufacturer’s instructions. The purity and quality of the DNA were verified in 0.8% agarose gels. Subsequently, the full-length 16S rRNA gene was amplified using the primers (27F, AGRGTTTGA TYNTGGCTCAG and 1492 R, TASGGHTACCTTGTTASGACTT). The purified PCR products were used to construct the single-read sequencing library on the PacBio platform (Biomarker-Technologies Co., China), following the manufacturer’s specifications. SMRT-Link v8.0 was used to correct the original subreads to obtain Circular Consensus Sequencing (CCS) sequence. The lima v1.7.0 software was used to identify the CCS sequence of different samples through the barcode sequence and remove the chimera (UCHIME v4.2), and obtain effective-CCS sequences. The generated datasets were analyzed using USEARCH v10.0. High-quality sequences were clustered as operational taxonomic units (OTUs) based on 97% similarity. BMK Cloud^1^ was used for Alpha diversity, Beta diversity, and microbial composition analysis to investigate differences in samples among groups.

### 2.7 Statistics analysis

All statistical analyses were conducted using SPSS software (version 26.0; IBM Corp., Armonk, NY, United States), with the results reported as mean ± Standard Error of Means (SEM). A one-way ANOVA test for multiple comparisons, followed by Dunnett’s *post-hoc* test, was employed to assess statistical significance between groups. Histograms were plotted using GraphPad Prism 8.0 (GraphPad, Inc. La Jolla, CA, United States). A probability value of *p* < 0.05 or *p* < 0.01 was considered statistically significant. A trend in significance was acknowledged for 0.05 < *p* < 0.10.

Alpha diversity indices (ACE and Shannon indices) of the samples were evaluated using QIIME2 2020 software, and the significance of differences was confirmed using the Wilcoxon rank sum test. After the alpha diversity analysis, Partialleast squares discriminant analysis (PLS-DA)was conducted with QIIME software based on OTU level.

## 3 Results

### 3.1 Growth performance

Throughout the entire experiment, the broiler chickens maintained good health. The effects of supplements on the growth performance of broilers were presented in Table 2. There were no significant differences in body weight (BW) of broilers at 1 and 28 d of age among groups (*p* > 0.05). Likewise, no significant differences were observed in ADG, ADFI, and F/G among groups from 1 to 28 d of age (*p* > 0.05). At 84 d of age, the BW in the GUE, Lac and GL groups significantly increased (*p* < 0.05) compared to the Con group, with the GL group showing the greatest improvement (*p* < 0.05). From day 29 to 84 and from day 1 to 84, the ADG of broilers in the GUE and Lac groups significantly increased (*p* < 0.05) compared to that in the Con group, while there was no significant difference in ADFI (*p* > 0.05). However, the GL group had a significant increase (*p* < 0.05) in both ADG and ADFI, along with a marked decrease (*p* < 0.05) in F/G compared to the Con group. Additionally, the F/G from day 1 to 84 in the Lac group was significantly lower (*p* < 0.05) than in the Con group.

**Table 2:** Effect of dietary GUE, Lac and their combination on the growth performance in broilers.

Items	Con group	GUE group	Lac group	GL group	SEM	*p*-value
Initial body weight, g	40.19	40.48	41.21	39.19	0.85	0.416
**d 1–28**						
BW, g	550.38	564.46	567.46	579.92	10.58	0.218
ADG, g/d	18.23	18.72	18.80	19.32	0.38	0.262
ADFI, g/d	48.30	48.10	48.06	48.23	1.51	0.999
F/G	2.65	2.58	2.57	2.51	0.10	0.829
**d 29–84**						
BW, g	3398.34^c^	3553.77^b^	3539.37^b^	3780.79^a^	27.21	0.019
ADG, g/d	51.10^c^	53.38^b^	53.12^b^	57.13^a^	0.188	0.042
ADFI, g/d	171.74^b^	177.07^ab^	172.97^b^	181.80^a^	2.48	0.035
F/G	3.36^a^	3.32^ab^	3.26^ab^	3.18^b^	0.05	0.041
**d 1–84**						
ADG, g/d	33.98^c^	41.81^b^	41.64^b^	44.54^a^	0.68	0.047
ADFI, g/d	130.60^b^	134.08^ab^	131.33^b^	137.27^a^	2.21	0.017
F/G	3.27^a^	3.21^ab^	3.15^bc^	3.08^c^	0.09	0.032

GUE, *Glycyrrhiza uralensis* extract; Lac, *Lactobacillus acidophilus*; GL, GUE and Lac; SEM, standard error of means. BW, body weight; ADG, average daily gain; ADFI, average daily feed intake; F/G, feed/gain. Values with the same or no letter superscripts in the same row mean no significant difference (*p* > 0.05), while with different letter superscripts mean significant difference (*p* < 0.05).

### 3.2 Intestinal antioxidant and sIgA

The effects of GUE, Lac and their combination on intestinal antioxidant capacity and sIgA content of broilers were presented in Table 3. In comparison to the Con group, the GUE, Lac and GL groups exhibited a significant increased SOD activity and a significant decreased MDA content (*p* < 0.05) in the jejunum mucosa of broilers at 28 and 84 d of age; the GSH-Px activity in broilers at 28 and 84 d of age in GUE and GL groups, and in broilers at 28 d of age in Lac group, significantly increased (*p* < 0.01). Additionally, a significantly higher SOD activity in broilers at 28 d of age was observed in the GL group compared to the GUE and Lac groups (*p* < 0.01). There was no significant difference in the sIgA content in the jejunum mucosa of broilers at 28 d of age among the groups (*p* > 0.05). However, the GUE, Lac and GL groups exhibited a significant increase of sIgA content at 84 d of age compared to the Con group (*p* < 0.01). Additionally, the sIgA content in the GL group was significantly higher than in the GUE group or Lac group (*p* < 0.05).

**Table 3:** Effect of dietary GUE, Lac and their combination on intestinal antioxidant indexes and sIgA content in broilers.

Items	Con group	GUE group	Lac group	GL group	SEM	*p*-value
**28 d of age**						
SOD, U/g	130.94^c^	150.32^b^	149.03^b^	178.43^a^	4.80	0.001
GSH-Px, nmol/min/g	157.19^c^	188.07^a^	178.49^b^	195.59^a^	5.89	0.009
MDA, nmol/g	13.13^a^	8.54^b^	9.60^b^	8.68^b^	1.01	0.040
sIgA, μg/g	85.56	87.43	85.47	85.27	3.34	0.963
**84 d of age**						
SOD, U/g	237.62^c^	256.92^b^	285.59^a^	298.76^a^	7.34	0.007
GSH-Px, nmol/min/g	344.04^b^	381.63^a^	357.76^b^	393.75^a^	6.03	0.002
MDA, nmol/g	15.74^a^	12.31^b^	13.60^b^	11.71^b^	0.88	0.048
sIgA, μg/g	78.65^c^	96.44^b^	94.64^b^	107.37^a^	1.84	0.001

SOD, superoxide dismutase; GSH-Px, glutathione peroxidase enzyme; MDA, malondialdehyde. Values with the same or no letter superscripts in the same row mean no significant difference (*p* > 0.05), while with different letter superscripts mean significant difference (*p* < 0.05).

### 3.3 Variation in cecal microbiota diversity

#### 3.3.1 Variation in alpha diversity

In the microbiome analysis, a total of 256,857 original CCS sequences were obtained through full-length 16S rRNA gene amplification on 40 cecal content samples using the PacBio platform. On average, 6,517 CCS sequences were generated in broilers at 28 d of age, with at least 4,400 CCS sequences for each sample. Similarly, an average of 6,326 CCS sequences were obtained in broilers at 84 d of age, with at least 4,074 CCS sequences for each sample. Following size filtering, quality control and chimera removal, a total of 199,146 high-quality sequences were retained, including 104,911 sequences at 28 d of age and 94,235 at 84 d of age.

**Figure 1:**
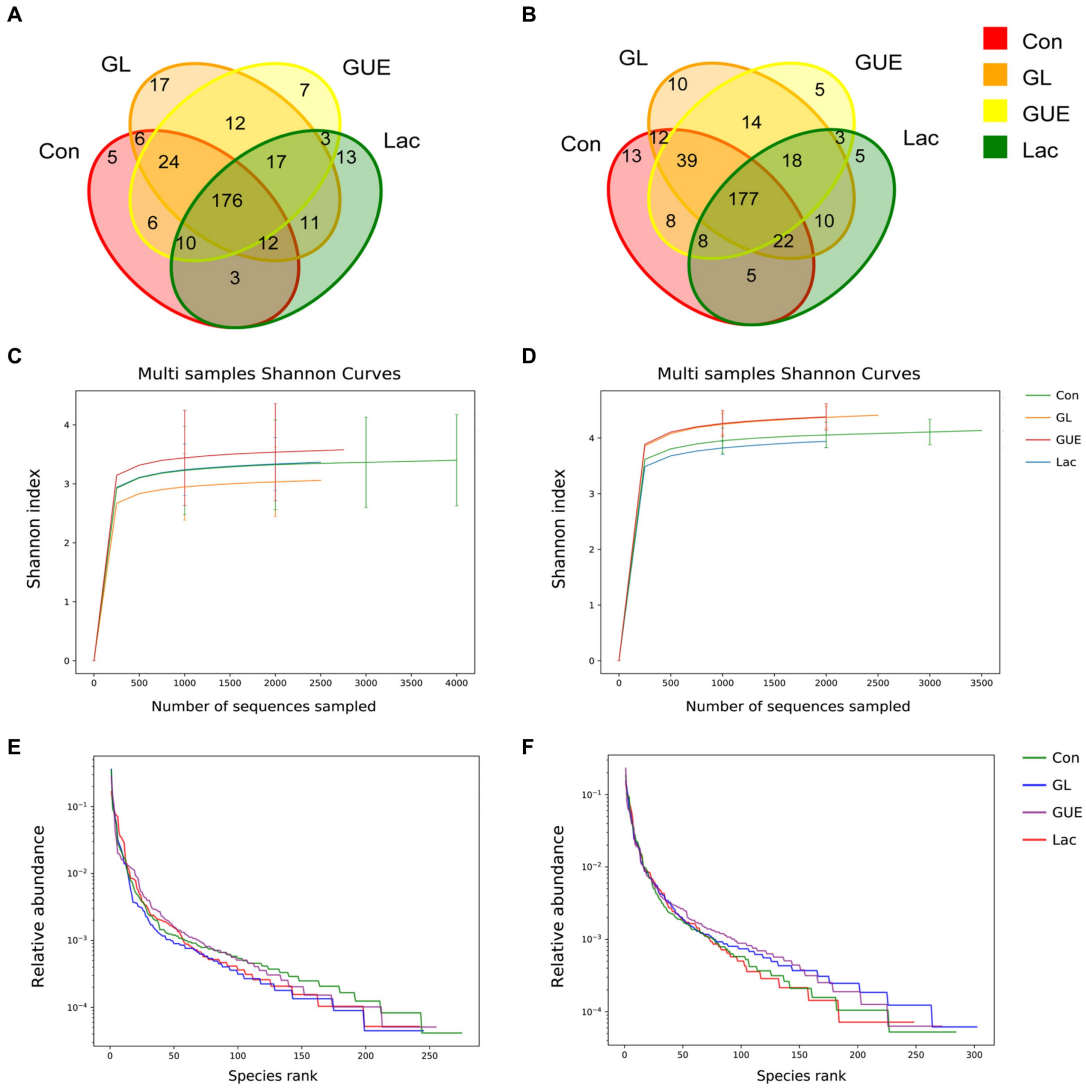
The Venn diagram, Shannon curves and Rank abundance curve of OTUs. **(A,B)** The Venn diagrams at 28 and 84 d of age. **(C,D)** The Shannon curves at 28 and 84 d of age. **(E,F)** The Rank abundance curve at 28 and 84 d of age.

At a 97% sequence similarity threshold, 322 OTUs were identified in samples from 28-d-old broilers in Con, GUE, Lac, and GL groups, with 5, 7, 13, and 17 unique OTUs, respectively (Figure 1A). At 84 d of age, 349 OTUs were identified in the four groups, with 13, 5, 5 and 10 unique OTUs, respectively (Figure 1B). The Shannon curves and the Rank abundance curve of cecal samples indicated that the sample size was reasonable, and the sequencing depth was sufficient for all samples based on a saturated trend (Figures 1C–F).

**Figure 2:**
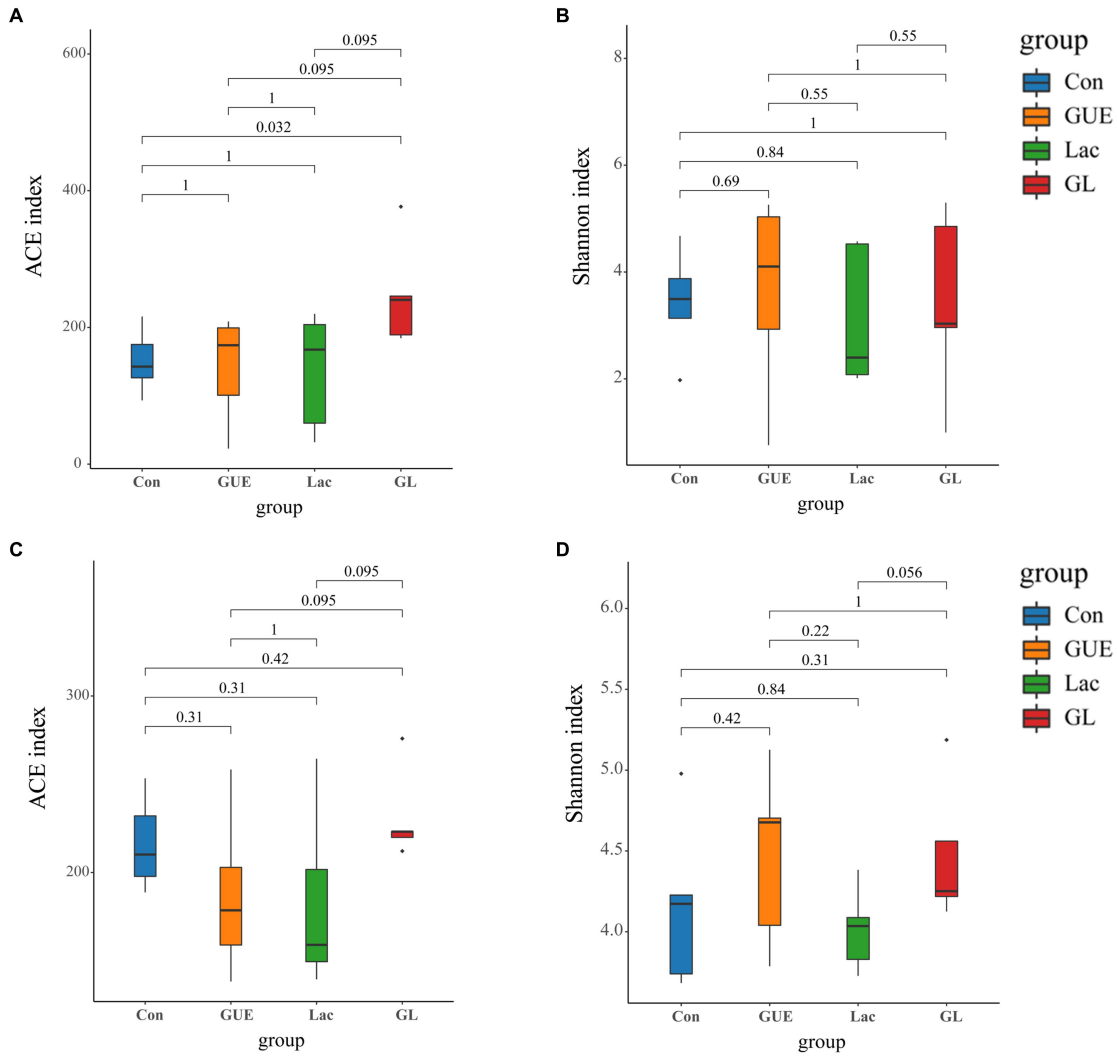
Alpha diversity of the microbiome residing in the cecal contents of broilers. **(A,B)** ACE and Shannon index in 28-d-old broilers. **(C,D)** ACE and Shannon index in 84-d-old broilers.

The alpha diversity index serves as an indicator of the species richness and diversity within individual samples (29). To assess the alpha diversity of the samples, the ACE and Shannon indices were determined. At 28 d of age, there was no significant difference in the Shannon index among the groups (*p* > 0.05; Figure 2B). However, the ACE index in the GL group was significantly higher than in the Con group (*p* < 0.05; Figure 2A). In comparison with the GUE or Lac groups, there was a tendency to increase in the ACE index in the GL group (*p* = 0.095; Figure 2A). At 84 d of age, the ACE index (*p* = 0.095) and Shannon index (*p* = 0.056) showed a tendency to increase in the GL group compared to the GUE group or Lac group (Figures 2C,D).

#### 3.3.2 Variation in beta diversity

**Figure 3:**
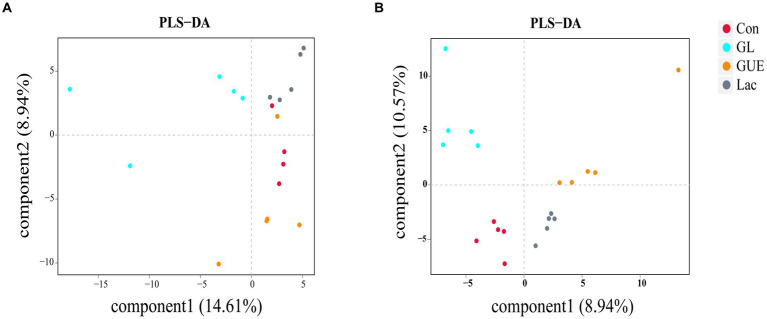
Beta diversity of the microbiome residing in the cecal contents of broilers. **(A,B)** Partial Least Squares Discriminant Analysis (PLS-DA) biplot at 28- and 84-d-old broilers.

Beta diversity, a measure of variance in taxa composition between sampling sites (30), was visualized by plotting the distances between samples on Partial Least Squares Discriminant Analysis (PLS-DA) biplot. At 28 d of age, there was no significant separation observed between different treatment groups, and the distribution of samples within each group was discrete (Figure 3A). At 84 d of age, the intestinal microbial community exhibited a distinct separation among the Con, GUE, Lac, and GL groups, with samples clustering within each group (Figure 3B).

### 3.4 Variation in cecal microbiota composition

#### 3.4.1 Phylum level

**Figure 4:**
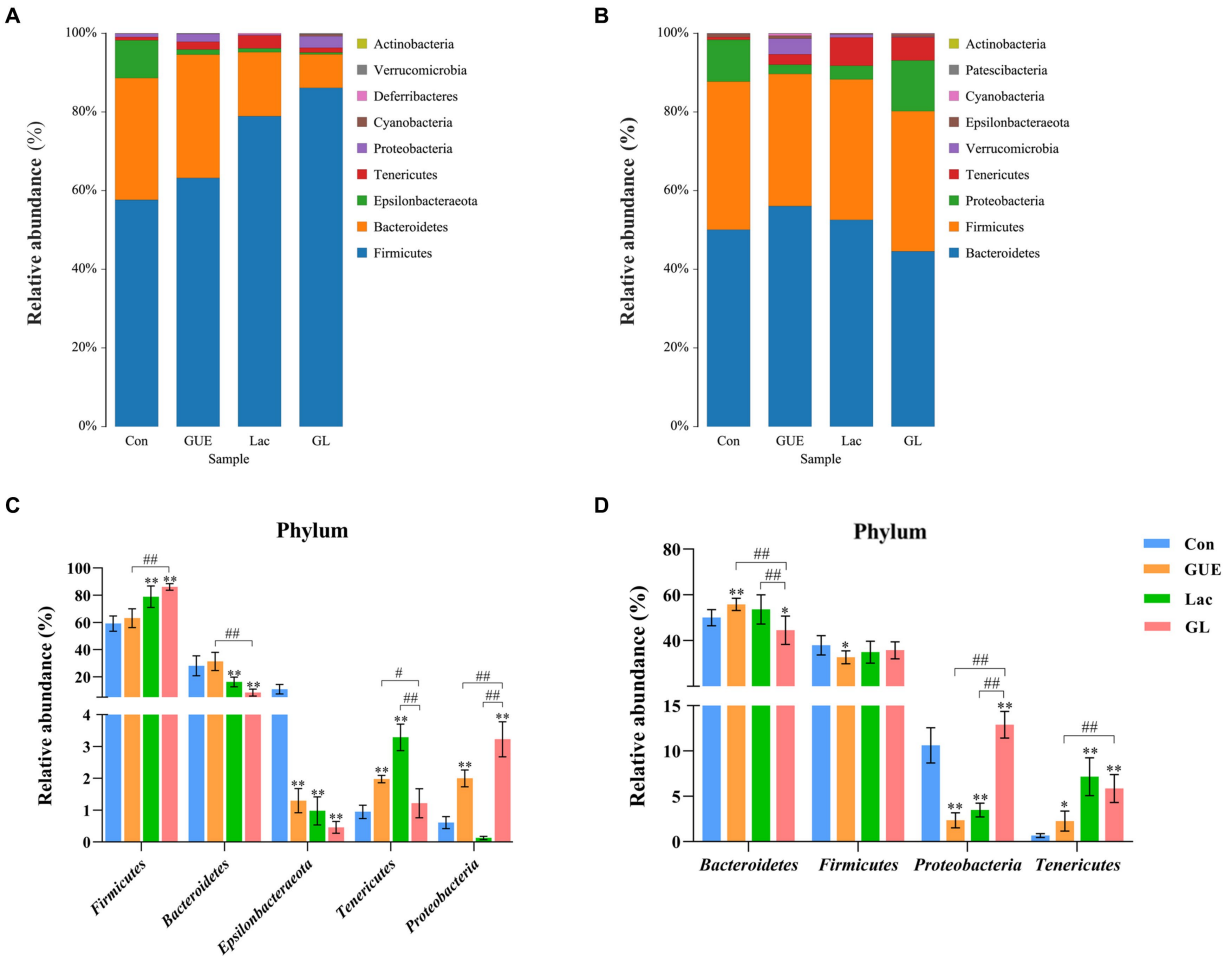
The relative abundance taxa of cecal microbiota in broiler chickens at the phylum level. **(A,B)** Relative abundance taxa at 28 and 84 d of age. **(C,D)** Relative abundance difference analysis of cecal bacterial species at the phylum level at 28 and 84 d of age, respectively. “*” indicated statistically significant difference from the Con group (*p < 0.05 and **p < 0.01). “#” indicated a statistical difference between the two groups (^#^*p* < 0.05 and ^##^*p* < 0.01).

The relative abundance of microbial composition at the phylum level was depicted in Figure 4. At 28 d of age, cecal samples in the Con, GUE, Lac, and GL groups were primarily dominated by five bacterial phyla: *Firmicutes* (59.24, 63.21, 78.93, and 86.13%, respectively), *Bacteroidetes* (28.16, 31.37, 16.27, and 8.52%, respectively), *Epsilonbacteraeota* (10.97, 1.30, 0.98, and 0.46%, respectively), *Tenericutes* (0.95, 1.98, 3.29, and 1.22%, respectively) and *Proteobacteria* (0.61, 2.00, 0.13, and 2.93%, respectively) (Figure 4A). Compared to the Con group, the relative abundance of *Tenericutes* and *Proteobacteria* significantly increased, while that of *Epsilonbacteraeota* significantly decreased in the GUE group. Similarly, the relative abundance of *Firmicutes* and *Tenericutes* significantly increased, while that of *Bacteroidetes* and *Epsilonbacteraeota* significantly decreased in the Lac group. In the GL group, the relative abundance of *Firmicutes* and *Proteobacteria* significantly increased, and that of *Bacteroidetes* and *Epsilonbacteraeota* significantly decreased (*p* < 0.05) (Figure 4C). Moreover, the relative abundance of *Firmicutes* and *Proteobacteria* significantly increased, while that of *Bacteroidetes* and *Tenericutes* significantly decreased in the GL group compared to the GUE or Lac group (*p* < 0.05) (Figure 4C).

At 84 d of age, cecal samples in the Con, GUE, Lac, and GL groups were dominated by four bacterial phyla: *Bacteroidetes* (50.06, 56.07, 52.55, and 44.55%, respectively), *Firmicutes* (37.68, 33.59, 35.73, and 35.65%, respectively), *Proteobacteria* (10.63, 2.36, 3.48, and 12.90%, respectively) and *Tenericutes* (0.66, 2.65, 7.17, and 5.87%, respectively) (Figure 4B). Compared to the Con group, the relative abundance of *Bacteroidetes* and *Tenericutes* significantly increased, while that of *Firmicutes* and *Proteobacteria* significantly decreased in the GUE group. Similarly, the relative abundance of *Tenericutes* significantly increased, while that of *Proteobacteria* significantly decreased in the Lac group. In the GL group, the relative abundance of *Proteobacteria* and *Tenericutes* significantly increased, while that of *Bacteroidetes* significantly decreased (*p* < 0.05) (Figure 4D). Additionally, the relative abundance of *Proteobacteria* and *Tenericutes* significantly increased, and that of *Bacteroidetes* significantly decreased in the GL group compared with the GUE or Lac group (*p* < 0.05) (Figure 4D). Thus, at the bacterial phylum level, the dietary supplements had a significant impact on the cecal microbial composition of broilers.

#### 3.4.2 Genus level

**Figure 5:**
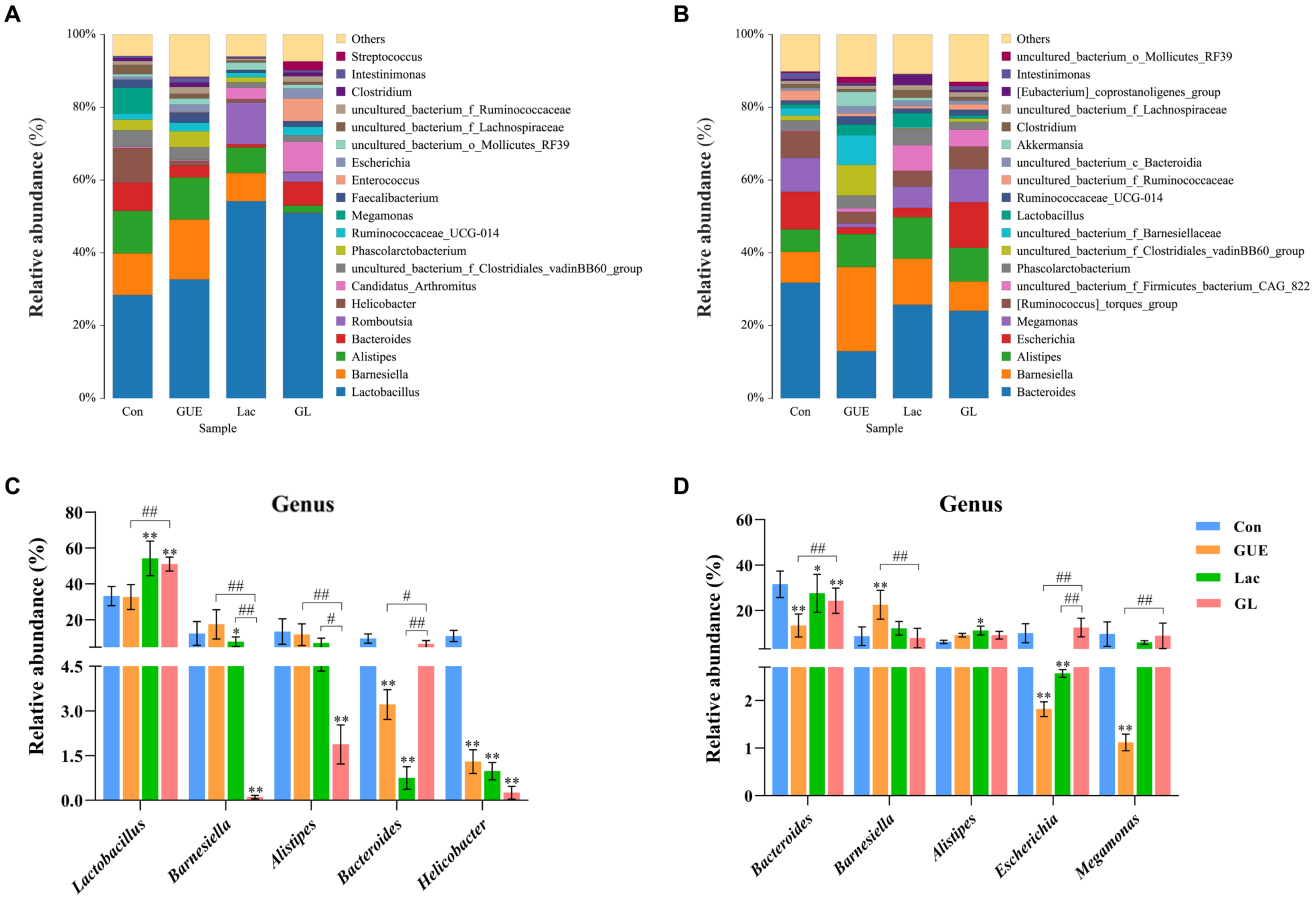
The relative abundance of cecal microbiota at the genus level. **(A,B)** Relative abundance taxa at 28 and 84 d of age. Relative abundances in the top 20 taxa were shown, and other taxon were combined as “Others.” **(C,D)** Relative abundance difference analysis of cecal bacterial species at the genus level at 28 and 84 d of age, respectively. “*” indicated statistically significant difference from the Con group (*p < 0.05 and **p < 0.01). “#” indicated a statistical difference between the two groups (^#^*p* < 0.05 and ^##^*p* < 0.01).

The relative abundance of the microbial composition at the genus level was showed in Figure 5. At 28 d of age, cecal samples in the Con, GUE, Lac and GL groups were primarily dominated by five bacterial genera: *Lactobacillus* (33.23, 32.67, 54.19, and 50.98%, respectively), *Barnesiella* (13.01, 16.47, 7.74, and 0.12%, respectively), *Alistipes* (5.56, 11.58, 7.03, and 1.88%, respectively), *Bacteroides* (9.49, 3.22, 0.88, and 6.51%, respectively) and *Helicobacter* (10.95, 1.30, 0.98, and 0.23%, respectively) (Figure 5A). Among them, the relative abundance of *Alistipes* in the GUE group and *Lactobacillus* in Lac and GL groups was higher than in the Con group (*p* < 0.05), while the relative abundance of *Helicobacter*, *Bacteroides* and *Barnesiella* was lower in the GUE, Lac and GL groups than in the Con group (*p* < 0.05) (Figure 5C). Additionally, the relative abundance of *Lactobacillus* and *Bacteroides* was higher, and that of *Barnesiella* and *Alistipes* was lower in the GL group than in the GUE or Lac group (*p* < 0.05) (Figure 5C). The relative abundance of other genera in the top 20 taxa varied among groups.

At 84 d of age, the most abundant taxa in the Con, GUE, Lac, and GL groups were *Bacteroides* (31.75, 12.93, 25.71, and 24.04%, respectively), *Barnesiella* (8.54, 23.15, 12.67, and 8.10%, respectively), *Alistipes* (6.15, 9.07, 11.40, and 9.24%, respectively), *Escherichia* (10.32, 1.82, 2.57, and 12.52%, respectively) and *Megamonas* (9.33, 1.12, 5.91, and 9.17%, respectively) (Figure 5B). These main bacterial genera showed significant differences among different treatment groups (*p* < 0.05) (Figure 5D). Additionally, the remaining bacterial genera also exhibited differences in relative abundances, such as *[Ruminococcus]_torques_group*, *Phascolarctobacterium* and *Lactobacillus*. In summary, the difference in supplements also affected the relative abundance of broiler cecal microbiota at the genus level.

#### 3.4.3 Species level

**Figure 6:**
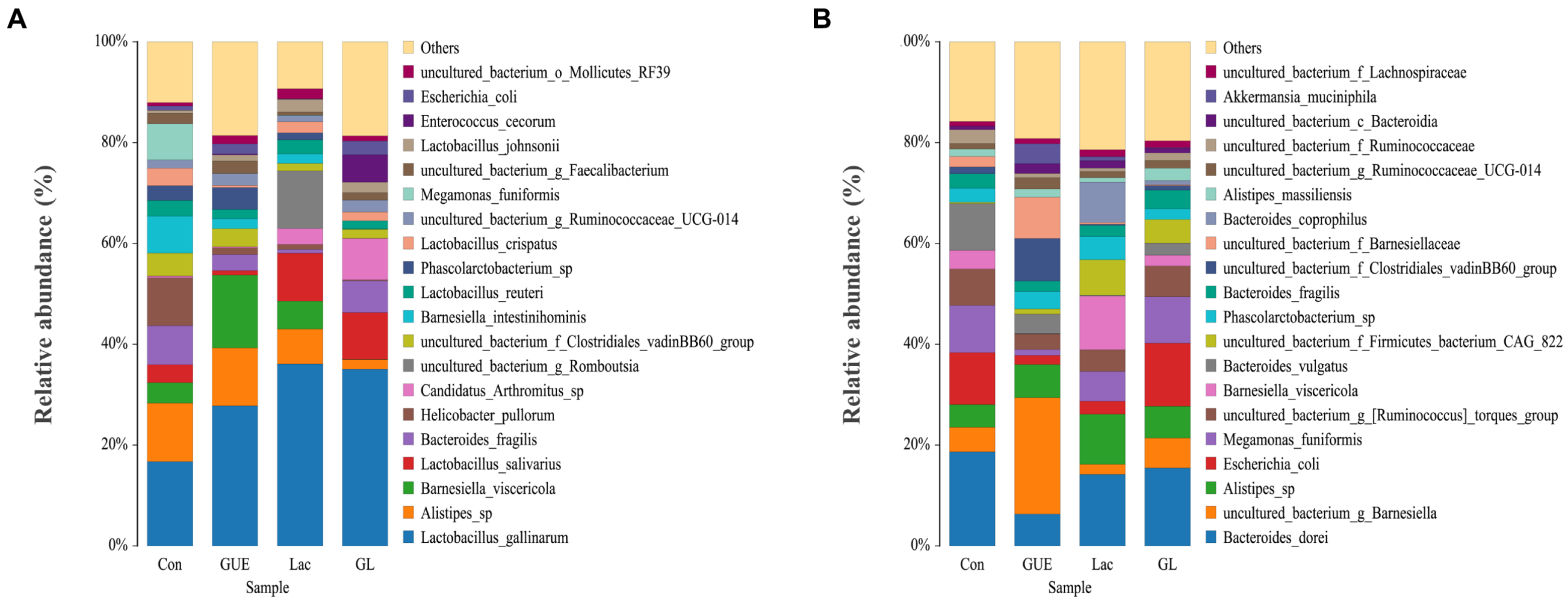
The relative abundance taxa of cecal microbiota in broiler chickens at the species level. **(A,B)** Relative abundance taxa at 28 and 84 d of age. Relative abundances in the top 20 taxa were shown, and other taxon were combined as “Others”.

The relative abundance of the microbial composition at the species level was presented in Figure 6. At 28 d of age, cecal samples in the Con, GUE, Lac, and GL groups was dominated by *Lactobacillus_gallinarum*, *Alistipes_sp., Barnesiella_viscericola*, *Lactobacillus_salivarius*, *Bacteroides_fragilis*, *Helicobacter_pullorum*, *Candidatus_Arthromitus_sp., Barnesiella_intestinihominis*, *uncultured_bacterium_g_Romboutsia* and *Lactobacillus_reuteri* (Figure 6A). Notably, *Lactobacillus_gallinarum* was the most dominant bacterium, accounting for more than 19% of the total microbial community detected. Compared to the Con group, the GUE, Lac and GL groups all significantly increased the relative abundance of *Lactobacillus_gallinarum* and significantly decreased that of *Helicobacter_pullorum*. Additionally, the GL group exhibited a significant increase in the relative abundance of *Bacteroides_fragilis* and *Candidatus_Arthromitus_sp.,* and a decrease in *Alistipes_sp., Barnesiella_viscericola* and *Barnesiella_intestinihominis* compared to the GUE or Lac groups (Supplementary Table S1).

At 84 d of age, the most abundant taxa in the Con, GUE, Lac, and GL groups were *Bacteroides_dorei*, *uncultured_bacterium_g_Barnesiella*, *Alistipes_sp., Escherichia_coli*, *Megamonas_funiformis*, *uncultured_bacterium_g*_[*Ruminococcus*]*_torques_group*, *Barnesiella_viscericola*, *Bacteroides_vulgatus*, *uncultured_bacterium_f_Firmicutes_bacterium_CAG_822* and *Phascolarctobacterium_sp* (Figure 6B). Compared with the Con group, the relative abundance of *Alistipes_sp* was significantly higher, while that of *Bacteroides_dorei* and *Bacteroides_vulgatus* was significantly lower in the supplemented groups. Additionally, the GL group displayed a notable increase in the relative abundance of *Megamonas_funiformis* and *uncultured_bacterium_g_[Ruminococcus]_torques_group*, along with a decrease in *Phascolarctobacterium_sp* compared to the GUE or Lac groups (Supplementary Table S2). The relative abundance of other species in the top 20 taxa also exhibited variability across groups.

### 3.5 Differences between groups in microbial diversity

**Figure 7:**
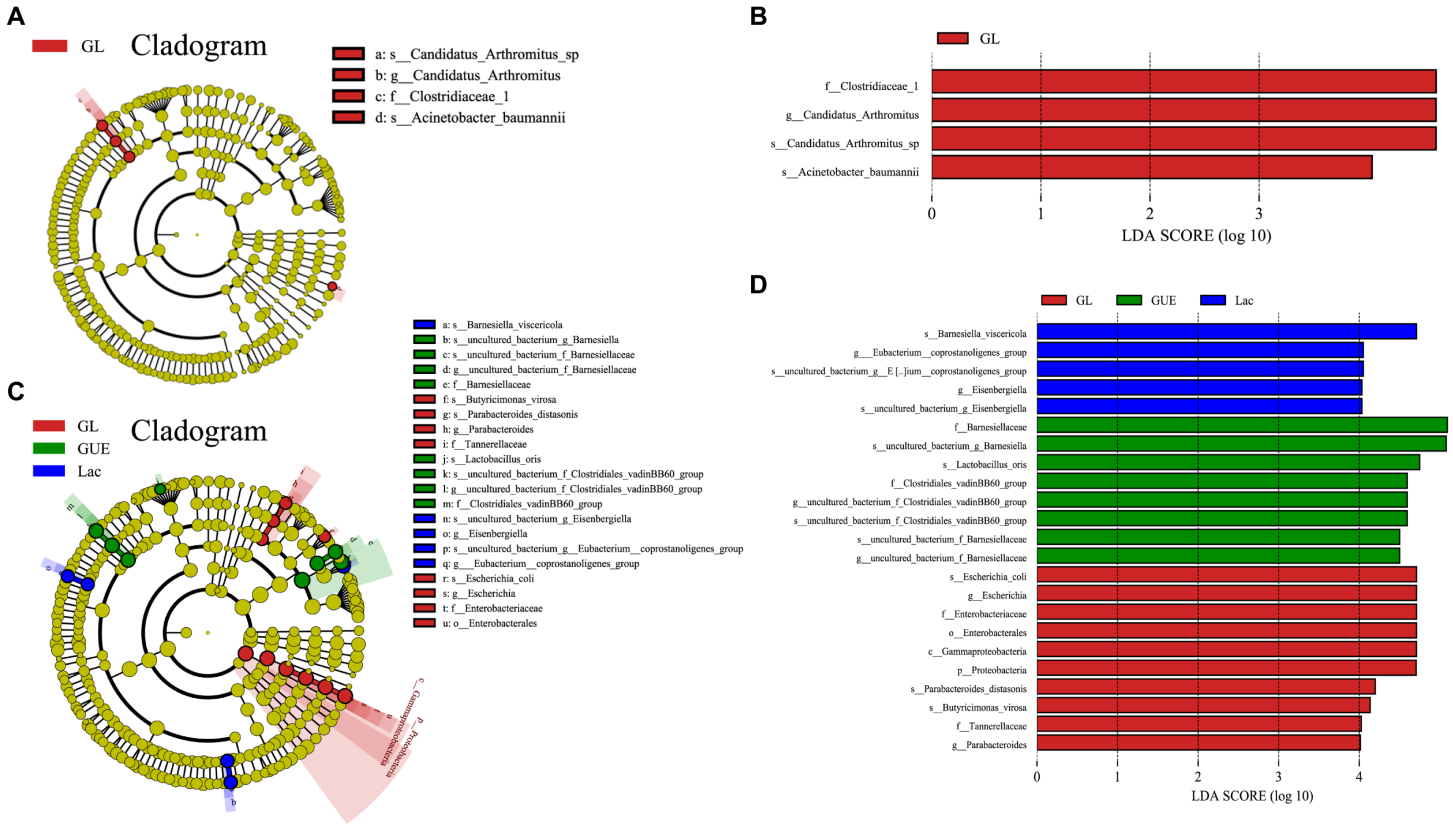
LEfSe taxonomic cladogram analysis and LDA score of cecal microbiota. **(A,B)** LEfSe taxonomic cladogram and LDA score at 28 d of age. **(C,D)** LEfSe taxonomic cladogram and LDA score at 84 d of age. The circles radiated from the center to the outer edges of the evolutionary branch map represent the classification level from the phylum to the species. The yellow nodes represent taxonomic units that show no significant differences between groups, and the size of the circle is positively correlated with relative abundance. Different colors indicate different groups. The length of the LDA histogram represents the LDA score, and the difference is significant when LDA > 4.0. p, phylum; c, class; o, order; f, family; g, genus; s, species.

The Linear discriminant analysis (LDA) combined LDA effect size (LEfSe) method was used to further analyze the differential marker species in the cecal samples in groups. At 28 d of age, 4 significant biomarkers were enriched in the GL group, namely *f_Clostridiaceae_1*, *g_Candidatus_Arthromitus*, *s_Candidatus_Arthromitus_sp.,* and *f_Moraxellaceae* (Figures 7A,B). At 84 d of age, 23 biomarkers were significantly enriched in the GUE, Lac and GL groups. Specifically, there were 8 species in the GUE group, including *f_Barnesiellaceae*, *s_Lactobacillus_oris*, *f_Clostridiales_vadinBB60_group*, 2 uncultured genera, and 3 unnamed species. The Lac group exhibited 5 species, including *s_Barnesiella_visceriricola*, *g_Eubacterium_coprostanoligenes_group*, *g_Eisenbergiella*, and 2 unnamed species. The GL group presented 10 species, including *s_Escherichia_coli*, *g_Escherichia*, *f_Enterobacteriaceae*, *o_Enterobacterales*, *c_Gammaproteobacteria*, *p_Proteobacteria*, *s_Parabacteroides_distasonis*, *s_Butyricimonas_virosa*, *f_Tannerellaceae*, and *g_Parabacteroides* (Figures 7C,D).

## 4 Discussion

### 4.1 Growth performance

Probiotics, certain medicinal plants and their extracts are being viewed as promising alternatives to in-feed antibiotics due to their unique functions, including preventing intestinal diseases, enhancing overall health and performance of poultry. Numerous studies have reported that dietary supplementation with *Lactobacillus acidophilus* (31) and *Lactobacillus plantarum* (27) could enhanced the growth performance of broilers by improving intestinal health. Additionally, supplementation with GUE maintained intestinal barrier integrity and improved growth performance of broilers by up-regulating the expression of the intestinal barrier function factors junctional adhesion molecule (JAM) and mucin 2 (MUC-2) (26), and increased weight gain in the late growth and throughout the growth period of broilers reared at high-density (32).

In the present study, it was shown that supplementation with GUE, Lac, and their combination significantly boosted the BW of broilers at 84 d of age, and increased the ADG from d 29 to 84 and throughout the entire experiment, while no significant impact on chickens aged 1 to 28 d were observed. Additionally, the combination GUE and Lac had a higher ADG and a lower F/G in broilers aged 1 to 84 days compared to their individual supplementation. These findings suggested that both GUE and Lac enhanced the growth performance of broilers, and the combination of GUE and Lac had a noticeable synergistic effect.

### 4.2 Intestinal antioxidant and sIgA

As a channel for animals to communicate with the external environment, the intestine is susceptible to oxidative stress caused by various factors. Zhang et al. (33) found that dietary *Glycyrrhiza* polysaccharide significantly increased the expression of NF-E2-related factor 2 (Nrf-2), SOD1 and CAT in the jejunum mucosa, and improved the antioxidant capacity of piglets. Probiotics have the effect of resisting oxidative stress in the intestine, and dietary *Lactobacillus plantarum* could regulate the expression and production of antioxidant enzymes, thereby activating antioxidant defense system (34). Our results indicated that supplementation with GUE and Lac increased the activity of antioxidant enzyme SOD and GSH-Px and reduced MDA content in the jejunum mucosa of 28- and 84-d-old broilers. Furthermore, there was a synergistic effect of their combination. These results suggested that both GUE and Lac could enhance intestinal antioxidant capacity by regulating antioxidant enzyme activity.

SIgA is present in the intestinal mucosa, protects the intestine from pathogens, and regulates the intestinal microbiota throughout animal development (35). The deficiency of sIgA could lead to incomplete intestinal barrier function, resulting in decreased productivity and even death of poultry (36). As the predominant Ig isotype in the intestinal mucosa, sIgA inhibited abnormal epithelial cell translocation and preventing excessive inflammatory responses induced by lipopolysaccharide of Shigella (37). The increase of sIgA secretion contributed to the improvement of intestinal mucosal health, which led to the enhanced nutrient absorption and improved growth performance in broilers (38).

The present study found that GUE increased sIgA levels in the jejunum mucosa, indicating a potential enhancement of intestinal health in broilers, consistent with findings from Wu et al. (39). Probiotics, defined as “living microorganisms,” are known for their immunomodulatory and sIgA-inducing effects (40, 41). Probiotic supplementation enhanced the intestinal sIgA response, leading to changes in the overall intestinal microbiota structure and its interaction with sIgAs, thereby affecting host health (42). In this study, we observed that Lac supplementation notably raised sIgA levels in the jejunum mucosa of broilers, indicating its potential to enhance the intestinal health. Moreover, the combination of GUE and Lac exhibited a stronger effect in enhancing sIgA secretion compared to their individual supplementation.

### 4.3 Variation in cecal microbiota diversity

In this study, the full-length 16S rRNA gene of the cecal microbiota in broilers was sequenced on the Sequel II platform. Although previous study showed that supplementation of probiotic *Lactobacillus* increased intestinal microbial diversity under heat stress condition (43), our study found that dietary Lac or GUE had no significant effect on the α diversity based on the OTU level of 28- and 84-d-old broilers. Interestingly, the combination of GUE and Lac exhibited a significant synergistic effect on the ACE index.

Beta diversity analysis primarily describes variations in composition among microbiota (44). We observed that dietary GUE, Lac and their combined supplementation did not affect the structure of the cecal microbiota in broilers at 28 d of age, but had a distinct separation among groups at 84 d of age. Microbiota colonization in the cecum is a dynamic process influenced by various factors such as diet, disease defense, and host interactions. There were differences in microbiota composition at different growth stages of broiler (45). Moreover, microbial colonization in the intestine is affected by genetic background of the host, too. *Liangfenghua* broiler, a medium-speed growth broiler (46), might require an extended period for the colonization and accumulation of microbiota in the intestine.

In addition, LEfSe analysis indicated that only GL supplementation enriched 4 differential bacteria markers at 28 d of age, which were mainly included in *Firmicutes* and *Proteobacteria*. At 84 d of age, GUE enriched 8 specific bacteria, primarily within *Bacteroidetes* and *Firmicutes*, Lac enriched 5 differential bacteria, mainly involved in *Bacteroidetes* and *Proteobacteria*, and GL enriched 10 differential bacteria, primarily within *Proteobacteria*, *Firmicutes* and *Bacteroidetes*. From the above, each supplement had its unique microbial populations, suggesting that supplementation with GUE and Lac increased the abundance of cecal specific microbiota, with a synergistic effect from their combination. Furthermore, these also reflected that the diversity of intestinal microbiota in broilers increased with age until it stabilized, consistent with the findings of beta diversity.

### 4.4 Variation in cecal microbiota composition

The species annotation results were analyzed to understand the growth-promoting mechanisms of GUE, Lac, and their combination through intestinal microbiota. In this study, *Firmicutes* and *Bacteroidetes* were the most predominant, followed by *Proteobacteriau* and *Tenericutes*, with only a few of bacterial sequences in other phyla, which was consistent with Liu et al.’s research on broiler (47). Similar results were found in the study on the intestinal microbiota composition of Muscovy ducks (48), indicating that the intestinal microbiota composition of poultry is relatively stable. This study also observed that *Firmicutes* and *Bacteroidetes* were the two most dominant phyla in the cecal microbiota of 28-d-old broilers, while *Bacteroidetes* and *Firmicutes* predominated in that of 84-d-old broilers. These suggests that as broilers grow, their intestinal microbiota becomes more diverse, eventually establishing a complex and dynamic microbiome (49, 50). Furthermore, *Firmicutes* and *Bacteroidetes* collectively impacted the host’s energy absorption and storage, and the *Firmicutes* to *Bacteroidetes* (F / B) ratio in the GIT influenced the host’s ability to obtain energy from feed (51). A higher F / B ratio was often linked to enhanced growth performance (52). In this research, dietary Lac and the combination of GUE and Lac increased the F / B ratio in 28-d-old broilers, suggesting that these supplements could improve the weight gain by influencing cecal microbiota composition in broilers. Qiao et al. (53) found that adding compound polysaccharides derived from *Astragalus* and *Glycyrrhiza* to the diet improved broiler weight gain by raising the intestinal F / B ratio. Additionally, GUE, Lac, and their combination increased the relative abundance of *Tenericutes* and decreased that of *Epsilonbacteraeota*. In the research by Yang et al. (54), lentinan ameliorated intestinal microbiota dysbiosis in high-fat diet mice by decreasing the abundance of *Epsilonbacteraeota*.

Based on the analysis of cecal microbial composition at the genus and species levels, we found that Lac and the combination of GUE and Lac significantly increased the abundance of *Lactobacillus* and *Lactobacillus gallinarum* in the cecum of broilers at 28 d of age. Gut-residing *Lactobacillus* not only communicated with each other but also with the intestinal epithelial lining to balance the intestinal barrier integrity and mucosal barrier defense, and ameliorate the host immune responses (55). Under production conditions, *Lactobacillus* could colonize the GIT of broilers soon after hatching, and their metabolic activity reduced the pH of the chyme, which helped prevent the growth of harmful intestinal bacteria (56). *L. gallinarum* is beneficial to intestinal health, modulating intestinal microbial composition, secreting protective metabolites (57), improving the intestinal absorption capacity (58), and inhibiting the colonization of *Salmonella* in GIT (59). Conversely, *Helicobacter* and *Escherichia* easily colonize the intestines of humans and animals, causing various diseases by modulating the production of intestinal inflammatory factors and disrupting intestinal mucosal permeability, damaging the intestinal barrier (60–62). In this study, both GUE and Lac reduced the abundance of *Helicobacter* and *Helicobacter pullorum* in 28-d-old broilers, and *Escherichia* and *Escherichia coli* in 84-d-old broilers. The combination of GUE and Lac had a synergistic effect on reducing the abundance of *Helicobacter* and *Helicobacter pullorum*.

In this study, we also observed the GUE, Lac and their combination remarkably decreased the abundance of genus *Bacteroides*, specifically *Bacteroides_fragilis* and *Barnesiella_intestinihominis* in 28-d-old broilers, and *Bacteroides_dorei* and *Bacteroides_vulgatus* in 84-d-old broilers. *Bacteroides* was positively correlated with serum inflammatory cytokines TNF-a, IL-1β, and IL-6, and dietary supplementation with *Glycyrrhiza* polysaccharides suppressed the proliferation of *Bacteroides* (1). *Bacteroides*, a Gram-negative anaerobic bacterium, primarily achieved mutualism with the host through utilizing polysaccharides (63). The composition of *Bacteroides* is diverse and complex, playing a crucial role in various metabolic activities in animals (64). Certain *Bacteroides* species have pathogenic potential, promoting intestinal bacterial penetration and causing diarrhea by producing enterotoxins on the surfaces of intestinal epithelial cells (65). While some members of the phylum are part of the normal GIT microbiota, they may cause opportunistic infections if the intestinal mucosal barrier integrity is disrupted (66). This infection is typically triggered by various microorganisms, with *B. fragilis* being the most prevalent, found in the GIT of healthy individuals and associated with anaerobic bacteremia (67). *B. dorei* can cause intestinal inflammation and is recognized as a bacterial pathogen. Lan et al. (68) reported that α-glycerol monolaurate regulated the cecal microbiota of broilers at late growth stage by reducing the relative abundance of opportunistic pathogens such as *B. dorei*. Additionally, Bamba et al. (69) proposed a potential association between *B. vulgatus* and ulcerative colitis. The results mentioned above indicated that GUE and Lac could regulate the balance of intestinal microecology by increasing beneficial bacteria and reducing harmful bacteria in the cecum of broilers.

## 5 Conclusion

In summary, dietary GUE and *L. acidophilus* improved the growth performance of *Liangfenghua* broiler chickens, especially during the growing-finishing period, and enhanced intestinal health. Moreover, they increased the richness and diversity of cecal microbiota, and modulated the balance of intestinal microecology by increasing beneficial bacteria and reducing harmful bacteria in the cecum. The combined use of GUE and Lac had synergistic effects on growth performance, intestinal health, and microbiota composition. These findings suggest that the combination of GUE and *L. acidophilus* as feed additives has better application prospects in the poultry industry. However, further study is needed to understand the mechanism by which the combined supplementation of GUE and Lac affects the intestinal microbiota.

## Data availability statement

The datasets presented in this study can be found in online repositories. The names of the repository/repositories and accession number(s) can be found in the article/Supplementary material.

## Ethics statement

The animal study was approved by Northwest Minzu University Animal Care and Use Committee. The study was conducted in accordance with the local legislation and institutional requirements.

## Author contributions

XL: Conceptualization, Data curation, Formal analysis, Investigation, Software, Validation, Writing – original draft, Writing – review & editing. JLi: Methodology, Resources, Validation, Writing – original draft. HY: Data curation, Methodology, Validation, Writing – original draft. YC: Software, Visualization, Writing – original draft. SL: Data curation, Methodology, Writing – original draft. SJ: Resources, Software, Writing – original draft. YZ: Formal analysis, Visualization, Writing – original draft. GZ: Conceptualization, Funding acquisition, Investigation, Supervision, Writing – review & editing. JLu: Conceptualization, Project administration, Supervision, Writing – review & editing.

## Footnotes

^1^http://www.biocloud.net/
