# Supplementary material for: Effect of supplementation with Glycyrrhiza uralensis extract and Lactobacillus acidophilus on growth performance and intestinal health in broiler chickens
Source: Front Vet Sci. 2024 Jul 18;11:1436807. doi: 10.3389/fvets.2024.1436807 (PMC11291472; doi:10.3389/fvets.2024.1436807)
Supplement: Supplementary file 1 [file Table_1.DOCX]

Supplementary Table 1

**Table S1.** Effect of dietary GUE, Lac and their combination on microbiota composition of the cecum at species level of broilers on day 28.

| **Item** | **Con group** | **GUE group** | **Lac group** | **GL group** | **SEM** | **Significant** |
| --- | --- | --- | --- | --- | --- | --- |
| *Lactobacillus_gallinarum* | 19.62^c^ | 27.79^b^ | 36.09^a^ | 35.05^a^ | 1.29 | *** |
| *Alistipes_sp* | 5.44^b^ | 11.47^a^ | 6.93^b^ | 1.87^c^ | 0.49 | *** |
| *Barnesiella_viscericola* | 5.03^b^ | 14.49^a^ | 5.54^b^ | 0.07^c^ | 0.49 | *** |
| *Lactobacillus_salivarius* | 4.35^b^ | 0.89^c^ | 9.51^a^ | 9.31^a^ | 0.39 | ** |
| *Bacteroides_fragilis* | 9.48^a^ | 3.17^c^ | 0.76^d^ | 6.28^b^ | 0.55 | ** |
| *Helicobacter_pullorum* | 10.95^a^ | 1.30^b^ | 0.98^b^ | 0.23^b^ | 0.39 | *** |
| *Candidatus_Arthromitus_sp* | 0.42^c^ | 0.22^c^ | 3.15^b^ | 8.18^a^ | 0.32 | ** |
| *Barnesiella_intestinihominis* | 7.95^a^ | 1.98^b^ | 1.88^b^ | 0.04^c^ | 0.45 | ** |
| *uncultured_bacterium_g_Romboutsia* | 0.01^b^ | 0.02^b^ | 11.46^a^ | 0.02^b^ | 0.30 | *** |
| *Lactobacillus_reuteri* | 3.60^a^ | 1.84^b^ | 2.81^a^ | 1.65^b^ | 0.25 | ** |

%

GUE, *Glycyrrhiza uralensis* extract; Lac, *Lactobacillus acidophilus*; GL, GUE and Lac; SEM, standard error of means. Values with the same or no letter superscripts in the same row mean no significant difference (*P* > 0.05), while with different letter superscripts mean significant difference (*P* < 0.05). "*" indicated statistically significant difference among groups (* *P* < 0.05, ** *P* < 0.01 and *** *P* < 0.001).
