# Supplementary material for: Effect of supplementation with Glycyrrhiza uralensis extract and Lactobacillus acidophilus on growth performance and intestinal health in broiler chickens
Source: Front Vet Sci. 2024 Jul 18;11:1436807. doi: 10.3389/fvets.2024.1436807 (PMC11291472; doi:10.3389/fvets.2024.1436807)
Supplement: Supplementary file 2 [file Table_2.DOCX]

Supplementary Table 2

**Table S2.** Effect of dietary GUE, Lac and their combination on microbiota composition of the cecum at species level of broilers on day 84.

%

| **Item** | **Con group** | **GUE group** | **Lac group** | **GL group** | **SEM** | **Significant** |
| --- | --- | --- | --- | --- | --- | --- |
| *Bacteroides_dorei* | 18.67^a^ | 6.30^c^ | 14.19^b^ | 15.46^b^ | 0.59 | *** |
| *uncultured_bacterium_g_Barnesiella* | 4.85^b^ | 23.11^a^ | 2.00^c^ | 5.93^b^ | 0.54 | *** |
| *Alistipes_sp* | 4.51^c^ | 6.58^b^ | 9.93^a^ | 6.32^b^ | 1.66 | ** |
| *Escherichia_coli* | 10.32^b^ | 1.82^c^ | 2.57^c^ | 12.52^a^ | 0.63 | *** |
| *Megamonas_funiformis* | 9.33^a^ | 1.12^c^ | 5.91^b^ | 9.17^a^ | 0.59 | *** |
| *uncultured_bacterium_g_[Ruminococcus]_torques_group* | 7.27^a^ | 3.14^b^ | 4.30^b^ | 6.13^a^ | 0.49 | ** |
| *Barnesiella_viscericola* | 3.68^b^ | 0.03^c^ | 10.67^a^ | 2.15^b^ | 0.60 | *** |
| *Bacteroides_vulgatus* | 9.30^a^ | 3.89^b^ | 0.16^c^ | 2.38^b^ | 0.58 | *** |
| *uncultured_bacterium_f_Firmicutes_bacterium_CAG_822* | 0.27^d^ | 1.00^c^ | 7.03^a^ | 4.69^b^ | 0.35 | ** |
| *Phascolarctobacterium_sp* | 2.77^c^ | 3.50^b^ | 4.58^a^ | 2.09^d^ | 0.36 | ** |

GUE, *Glycyrrhiza uralensis* extract; Lac, *Lactobacillus acidophilus*; GL, GUE and Lac; SEM, standard error of means. Values with the same or no letter superscripts in the same row mean no significant difference (*P* > 0.05), while with different letter superscripts mean significant difference (*P* < 0.05). "*" indicated statistically significant difference among groups (* *P* < 0.05, ** *P* < 0.01 and *** *P* < 0.001).
